# ACL status in arthroplasty patients, why not to preserve?

**DOI:** 10.1051/sicotj/2017042

**Published:** 2018-01-05

**Authors:** Ahmed Abdelbadie, Ahmed Ali Toreih, Mohamed Ahmed Radwan

**Affiliations:** 1 Department of Orthopedic Surgery and Trauma, Suez Canal University Hospitals Kilo 4.5 Ring Road 41111 Ismailia Egypt

**Keywords:** Bicruciate retaining, Arthroplasty, ACL retaining, Knee replacement

## Abstract

*Introduction*: Only 70–85% of patients that had total knee arthroplasty (TKA) are satisfied with their knees. The need for a near to normal knee kinematics is crucial and maybe the solution to their needs. Addressing the cruciate ligaments during surgery along with the extent of arthrosis may give a solution to this problem.

*Material and methods*: One hundred consecutive patients in whom a total knee arthroplasty was indicated and performed were prospectively documented. During the knee replacement surgery, the condition of the anterior and posterior cruciate ligaments and the degree of osteoarthritis (OA) in the medial and lateral compartments as well as in the patello-femoral joint were documented using the Outerbridge classification. The patients’ average age was 72.3 years, with the majority being female. In all patients, a total bi-compartmental knee replacement was indicated.

*Results*: Our results showed that in 78% of all patients the anterior, and in 98% the posterior cruciate ligament was still intact. Seventy-one percent of cases suffered from grade 4 medial osteoarthritis, 19% from grade 3 and 10% from grade 2. Thirty-six of patients suffered from grade 4 lateral osteoarthritis, 36% from grade 3, 24% from grade 2 and 4% from grade 1. Grade 4 patello-femoral osteoarthritis was present in 32% of all patients, grade 3 in 60% and grade 2 in 8% of all patients.

*Discussion*: The goal of arthroplasty is to approximate the function of a normal knee. The retention of the anterior cruciate ligament (ACL) allows for better knee, kinematics, improved proprioception, increased flexion and an overall improvement in knee function. The decreased constraint that is possible with retention of both cruciates may decrease implant stresses and improve the implant survivorship. The distribution of OA shows that the medial and patello-femoral compartments of the joint are primarily affected. This could also allow for a more conservative and patient-tailored prosthetic design.

## Introduction

For the evaluation of the success of total knee arthroplasty (TKA) criteria such as the 10-year survival rate, range of movement or specialized joint scores are widely used. These usually show good to excellent results in most of the studies. These objective evaluation criteria leave aside the subjective patient satisfaction, which is the ultimate goal of all orthopaedic procedures. The primary aim of knee replacement is relief of pain, and in this respect, arthroplasty is successful. However, when this goal is achieved early in the postoperative period, patients may revise their priorities and define the success of their treatment in terms of secondary goals, principally restoration of joint function. Both the Swedish registries as well as surveys conducted by different German health insurance companies have shown that individual patient satisfaction is much less than is to be expected from the literature. Numerous clinical reviews have shown that only 15–30% of patients are not satisfied with their outcome after TKA. Also, patients increasingly expect advances in surgical techniques and implant designs to enable them to return to their original functional status not only a painless sedentary activity. There may be different reasons for dissatisfaction. One possible cause could be the fact that most of the TKA designs will lead to partial or complete loss of the anterior and posterior cruciate ligaments (ACL and PCL). Various studies however show that the preservation of the ACL and PCL during uni- or bi-compartmental TKA improves the functional results [1–7].

In the clinical setting, it is often assumed that a majority of patients needing knee prosthesis have a deficient ACL anyway or the kinematics of arthroplasty will leave a malfunctioning ligament. Moreover, technical difficulties are anticipated with ACL retention with inconclusive clinical benefits as a result of lack of studies. In the recent literature, there is very little information about this topic [8].

The objective of the present study was to document the situation in the ACL and PCL and the degree of osteoarthritis (OA) in the different compartments of patients who receive TKA.

## Material and methods

In a consecutive prospective series, 100 patients on whom a bi-compartmental TKA was indicated were included in this study. During surgery, the condition of the cruciate ligaments was documented by the operating surgeon. The ACL or PCL was considered to be intact, if femoral and tibial insertion sites could be well defined, the intra-ligamentous substance was macroscopically intact and if the mechanical function tested by the Lachman’s test and anterior and posterior drawer tests was intact.

At the same time, the degree of OA in the medial and lateral compartments and the patello-femoral compartment (using the Outerbridge classification, Table 1) was documented.

**Table 1.** Outerbridge classification.

Grade 1: swelling and softening of articular cartilage, possibly with additional fissures on the surface.
Grade 2: a partial-thickness defect with fissures on the surface that do not reach subchondral bone or exceed 1.5 cm in diameter.
Grade 3: fissuring to the level of subchondral bone in an area with a diameter more than 1.5 cm.
Grade 4: exposed subchondral bone.

The patients’ average age was 72.3 years (range: 47–82 years), with the majority being female (female:male ratio = 66:34). All patients were admitted to hospital following the indication of a TKA.

## Results

In our patient’s population, 78% were ascertained to have structurally and functionally intact ACL. The proportion was much higher in female patients with 61 out of 66 patients (92%) than with male patients with 21 out of 34 (62%). With regard to PCL, this was judged to be intact in 98% of the patients.

**Figure 1.**
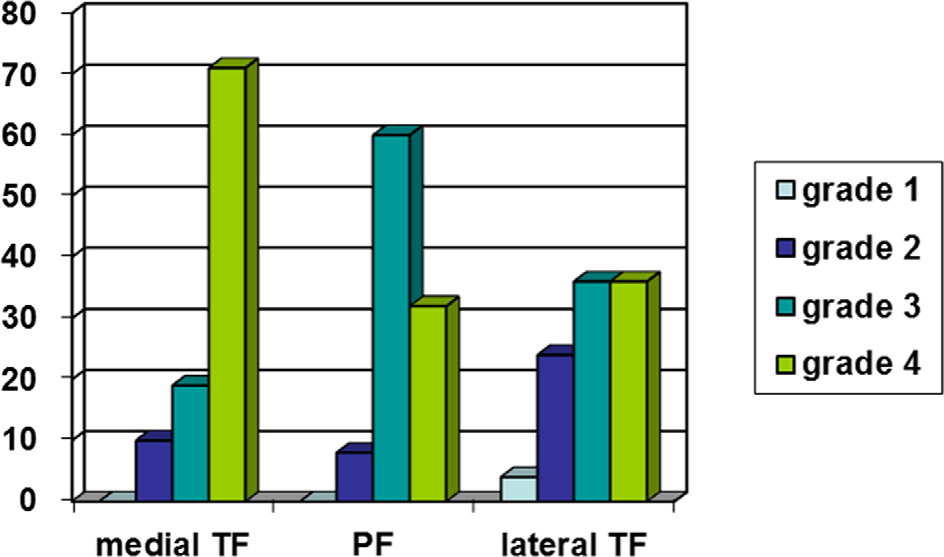
Osteoarthritis distribution in the different compartments according to Outerbridge classification.

In the medial compartment, 71% of cases suffered from grade 4 (using the Outerbridge classification) OA, 19% from grade 3 and 10% from grade 2. In the lateral compartment, 36% of patients suffered from grade 4 osteoarthritis, 36% from grade 3, 24% from grade 2 and 4% from grade 1. Grade 4 patello-femoral cartilage erosion was present in 32% of all patients, grade 3 in 60% and grade 2 in 8% of all patients (Figure 1).

## Discussion

There are a number of factors influencing the load pressure in the knee joint. These include joint geometry, the menisci, the tendons, the body weight and the tension of the muscles crossing the knee joint.

In arthroplasty settings, joint geometry is determined by the prosthetic design. Both of the menisci are removed in the TKA. Most of the knee replacement designs require the removal of the ACL, even if, as shown by our study, the majority of patients still have this ligament intact. The integrity of the ACL was also noted in different articles [9–11]. As shown below, the removal of the ACL subsequently has an influence on muscle tension and the muscles crossing the knee joint.

These muscles are responsible for reducing pressure on the knee joint. It is shown that the knee muscles absorb 3.6 times more energy than they generate [12]. It had already been established in the 1980’s that proprioceptive information originates from the cruciate ligaments [13, 14]. Gómez-Barrena et al. (1992) went on to prove an axonal transport of neurotransmitters from the cruciate ligament to the dorsal root ganglia [15].

Before that Solomonow et al. (1987) had been postulating a synergy between the ACL and the flexors, demonstrating a grade 1 reflex through the ACL receptors and a grade 2 reflex through the muscle and capsule receptors [16].

The arthroscopic stimulation of the ACL leads to sensitive evoked sensorimotor potentials in the cerebral cortex [17]. Mechanical load on the ACL leads to positive electromyographic (EMG) amplitude of the flexors [18]. Flexor and semitendinosus tendon EMG response on electrical stimulation of the ACL after 50–180 msec can be blocked by local anaesthetics [19].

There is therefore no doubt that the cruciate ligament uses its mechanoreceptor as a sensor able to convert mechanical load into afferent impulses. In this process, it would seem that a so-called feed-forward mechanism is used, as a direct reflex mechanism is not fast enough to compensate for the peak pressure – such as valgus stress in an accident – fast enough. In such a case, peak pressure is reached after 40–70 msec [20].

The above-mentioned reflex is not however fast enough, if it is only induced upon application of pressure [21]. Johansson was able to demonstrate a mechanism belonging to this so-called feed-forward system [22]. He showed that stretching the ACL using the gamma-motor neuron mechanism leads to an increase in the tone of the muscles crossing the knee joint.

Concerning muscle tension, a differentiation has to be made between an intrinsic muscle tension which is always present and which can be seen as an initial line of defence, and an extrinsic muscle tension stimulated by alpha and gamma motor neurons [23]. The latter can be modified by sensorimotor activity training. It is also interesting to note that, from a proprioceptive view of an ACL sufficiency any unilateral injury can be shown to cause a bilateral proprioceptive deficiency [24–26].

From a clinical point of view, it can be demonstrated that patients with an ACL rupture suffer from a major impairment in the sensorimotor activity of the affected and the contralateral knee joint. This can be demonstrated by the use of various test procedures such as active and passive angle reproduction tests. The question whether a defective ACL has an influence on the function of the prosthesis has up to now not received much attention in the literature. Fuchs et al. (2003) investigated the proprioceptive performance of patients with bilateral sledge prostheses [1]. The authors were able to demonstrate that the patients, after having had bicruciate-retaining arthroplasty, showed proprioception comparable with that in healthy test persons of the same age. Stiehl et al. (2000) performed *in vivo* fluoroscopic kinematic analyses using three-dimensional computer simulation [2]. They compared 16 prostheses where both the ACL and PCL had been retained with six prostheses where only the PCL had been retained. The authors were able to demonstrate that the prosthesis where only the PCL was retained had the worse kinematics.

Komistek et al. (2002) compared 15 prosthesis patients where the ACL had been retained with 15 patients where only the PCL had been retained [3]. They were able to demonstrate that patients retaining the ACL showed a kinematic pattern much more similar to that of a normal knee than patients with prosthesis where only the PCL was retained. Moro-oka et al. (2007) compared the kinematics of arthroplasty in patients where both ACL and PCL had been retained with those of patients where only the PCL had been retained [4]. This comparison showed that the retention of both ligaments was crucial for retaining normal knee behaviour. This was also demonstrated by Reider et al. (2007); early results in patients having undergone bicruciate-retaining TKA conclusively proved that this method led to normal knee joint kinematics [25].

Few clinical studies discussed the clinical survivorship of ACL-retaining prosthesis. Lack of enthusiasm, as many considered those prostheses as technically demanding and of limited clinical value, may be the reason for that. Buechel and Pappas [27] reported 91% 12-year survival in bicruciate-retaining in their series of only 21 patients. They conclude the prosthesis as equivalent as PCL substituting performance but they had few patients in the final follow-up.

Cloutier [28] published two patient series, the first one demonstrated a success rate of 96% at 10 years follow-up compared to 75% when the ACL was not retained. More interestingly, he concluded that it was technically easy to retain both cruciates in half of his patients. Also, he demonstrated a better flexion range. He did not use a dished tibial tray, which may be a reason for better clinical results when the ACL was retained.

In his second series, a total of 163 TKA in with cruciate-retaining designs were assessed in a prospectively [29]; 107 knees were followed for an average of 10 years. The ACL was considered relatively normal in 96 knees and partly degenerated in 67 knees. The result was excellent or good in 97% of patients and the survival rate, with revision as the end point, was 95% ±2% at an average of 10 years postoperatively. The average knee score was 91 and the average functional score was 82.

Jenny and Jenny [30] noted that the mean operative time, implant positioning and revision rates were the same in their series of patients on whom 32 ACL-retaining and 93 ACL-sacrificing TKAs were included. This study did not support the suggestion that technical difficulties may be increased in ACL retention during TKA.

Pritchett [31] published an interesting study of 50 patients with bilateral TKA, one side bicruciate retaining and other side only PCL retaining. Postoperative flexion averaged 119° in both groups and all patients had full extension. All knees that retained both cruciate ligaments were stable, whereas six knees in the posterior cruciate-only group had sagittal plane instability of more than 1 cm with anterior drawer test. The patients’ opinion about their knees was assessed. Seventy percent preferred the bicruciate-retaining knee and only 10% preferred the PCL-retaining knee; 20% did not express a preference. In addition, 29 of the 50 patients stated that they used their bicruciate-retaining TKA as their lead leg during stair-climbing, compared with only seven for the PCL-only-retaining knee.

More recently, Pritchett published his experience with 23-year follow-up of 489 patients who underwent ACL sparing TKA. He reported a survivorship of 89% with only 22 revisions due to polyethylene wear [32].

In our study, we also found that a large proportion of patients had patello-femoral OA in addition to medial compartment OA. Similar observations were made by Duncan et al. (2006), who found some 40% of patients having combined tibio-femoral and patello-femoral OA. Twenty-four percent of the 819 examined patients, all over 50 years of age, had isolated patello-femoral OA [33]. The question of whether patello-femoral OA led to clinical symptoms was examined by Englund and Lohmander [34]. They were able to demonstrate that in different activities patients with tibio-femoral and patello-femoral OA had more clinical symptoms than patients with just tibio-femoral OA [34]. This was especially the case when sports activities were involved. This suggestion could be a rationale for a more conservative bicruciate sparing uni-compartment arthroplasty alone or in conjunction with patella-femoral component.

## Conclusion

A significant number of patients needing a TKA still have an intact ACL. The goal of TKA is to approximate the function of a normal knee. The retention of the ACL allows for better knee, kinematics, improved proprioception, increased maximum flexion and an overall improvement in the knee function. The low constraint that is possible with the presence of both cruciates may decrease implant stresses and improve the longevity of these implants.

The distribution of OA shows that the medial and patello-femoral compartments of the joint are primarily affected. This could also allow for a more conservative and patient-tailored prosthetic design.

## Conflict of interest

Authors certify that they have no financial conflict of interest (e.g., consultancies, stock ownership, equity interest, patent/licensing arrangements, etc.) in connection with this article.
